# Developmental induction of human T-cell responses against *Candida albicans* and *Aspergillus fumigatus*

**DOI:** 10.1038/s41598-018-35161-5

**Published:** 2018-11-15

**Authors:** Katrin Vogel, Mandy Pierau, Aditya Arra, Karen Lampe, Dirk Schlueter, Christoph Arens, Monika C. Brunner-Weinzierl

**Affiliations:** 1 Department of Experimental Paediatrics, University Hospital, Otto-von-Guericke University, Magdeburg, Germany; 2 Department of Otorhinolaryngology, Head and Neck Surgery, University Hospital, Otto-von-Guericke University, Magdeburg, Germany; 3 Institute of Medical Microbiology and Hygiene, Otto-von-Guericke-University, Magdeburg, Germany; 4 Organ-specific Immune Regulation, Helmholtz Centre for Infection Research, Braunschweig, Germany; 5 Health Campus Immunology, Infectiology and Inflammation, Otto-von-Guericke University, Magdeburg, Germany

## Abstract

The origin of human T-cell responses against fungal pathogens early in life is not clearly understood. Here, we show that antifungal T-cell responses are vigorously initiated within the first years of life against lysates and peptides of *Candida albicans* or *Aspergillus fumigatus*, presented by autologous monocytes. The neonatal responding T-cell pool consists of 20 different TCR-V_β_ families, whereas infant and adult pools display dramatically less variability. Although we demonstrate no bias for anti-fungal IL-4 expression early in life, there was a strong bias for anti-fungal IL-17 production. Of note, only T-cells from neonates and infants show an immediate co-expression of multiple cytokines. In addition, only their T-cells co-express simultaneously transcription factors T-bet and RORγt in response to fungi and subsequently their target genes IL-17 and IFNγ. Thus, T-cells of neonates and infants are predetermined to respond quickly with high plasticity to fungal pathogens, which might give an excellent opportunity for therapeutic interventions.

## Introduction

Humans are exposed to fungi such as *Aspergillus fumigatus* (*A*. *fumigatus*) and *Candida albicans* (*C*. *albicans*) e.g. by inhaling spores or by ingesting contaminated food. Especially fungi such as *C*. *albicans* have co-evolved with humans and even colonize their body surfaces. Colonization by Candida yeast is thought to occur at an early age, with the organism being acquired during passage through the birth canal, nursing, or from food. An opportunistic infection by *C*. *albicans* is initiated rarely, but in particularly frequent from 4 weeks to 9 months of age. Most individuals affected from fungal pathologies are immunosuppressed, patients with disrupted mucosal barriers, patients taking multiple antibiotics, and young infants. Surprisingly, overall mortality of pediatric candidemia is high and did not improve over the past decade^1^.

Likely, fungi-exposure induces long lasting adaptive immune responses^2–4^ and especially the CD4 T cell compartment of the adaptive immune response is critically involved in efficient fungal defence, as demonstrated in HIV patients having low CD4 T cell counts^5^. Naïve CD4^+^ T cells differentiate when encountering their antigen presented by APC, into different T-helper (Th) subsets, i.e. Th1, Th2, Th9, Th17, Th22, which have signatory cytokine expression^6^. Th1 cells produce the cytokines IL-2, IFNγ, and TNFα and are decisive for host defence against intracellular pathogens^7,8^. For Th2 cells, responses are associated with the secretion of cytokines such as IL-4, IL-5, IL-10, IL-13, and IL-24. In response to IL-1β, IL-6, and TGFβ^9,10^ Th17 cells are differentiated and maintained that produce IL-17. Furthermore, peripheral Th cells such as Th17 cells show to some extend flexibility meaning that they gain characteristics of other lineages e.g. Th17 cells are able to become Th1-like cells^11,12^. T cell responses to *C*. *albicans* have been described as a finely tuned balance between Th1, Th17 and Treg subsets^13^. The clearance of infections caused by *C*. *albicans* on mucosal surfaces was shown to be driven by Th17 responses^14^, indeed, whereas Th1 and Th17 cells are regarded to be the cell types in providing immune response to oral and dermal candidiasis^2,15^. In regard to *A*. *fumigatus*, specific effector/memory CD4 T cells derived from peripheral blood of healthy adult donors displayed a Th1 phenotype^16^ and *A*. *fumigatus*-expanded Th1 cells are currently being tested for adoptive therapy upon stem cell transplantation^17^ (Trail EudraCT 2013-002914-11). However, lung-derived *Aspergillus*-specific T cells of the effector/memory subpopulation show a Th17 phenotype with notably production of IL-17^18^, implicating the involvement of a complex adaptive immune response in clearance of fungal pathogens.

Besides recognizing that the immune system in the neonatal and infant period is distinct from the adult one^19–21^, not much is known about its abilities to defend pathogens antigen-specifically. At birth, being suddenly exposed to antigens and pathogens of the environment, an immediate defence is required while inflammation is kept low in order to protect developing tissues. Recently, a general characteristic production of Th2 cytokines by human neonatal T cells has been reported, whereas, Th1 responses were reported to be weaker^22,23^. In addition, neonatal T cells also produce high frequencies of CXCL8 upon stimulation^24,25^. In addition, especially IL-17 is enhanced produced in term and preterm infants compared to adults^26^. Supporting the importance of IL-17, mutations in the IL17R causes chronic mucocutaneous candidiasis starting at 6 month of age^27^. Although rare, data on antigen-specific responses on cord blood T cells is arising using fungal-stimulation combined with polyclonal expansion procedures^28–31^, which gives evidence that naïve T cells at birth are able to respond to *C*. *albicans*. Taken together, knowledge on initiation of specific, antifungal T-cell responses of neonates, infants, and even children are limited and, thus, has to be complemented if we are to learn how early-life adaptive immune responses affect paediatric and in the long run - adult health.

## Materials and Methods

### Samples

PBMCs were obtained from leukocyte reduction filters (Sepacell RZ-2000; Asahi Kasaei Medical) supplied by the Institute of Transfusions Medicine and Immunohaematology at the University Hospital of Magdeburg. Cord blood samples were obtained from umbilical cord veins immediately after birth from the Women’s Clinic of the University Hospital of Magdeburg. The blood and adenoids were obtained from children (age 0.5–12 years of age) suffering from adenoid hypertrophy through surgical excision and supplied by the Department of Otolaryngology of the Medical University Hospital in Magdeburg. All infants, children, and adults had no history of recurrent infections or inflammations and within the last 4 weeks no cold/flue or intake of antibiotics. At the time of surgery or donation, donors were clinically free of infection. The study had formerly been approved by the Clinical Research Ethics Board of the University of Magdeburg (certificates 06/11, 79/07 and 26/12), and all donors and parents provided written informed consent in accordance with the declaration of Helsinki.

### Cell purification and cell culture

Mononuclear cells were obtained from cord blood (CB), peripheral blood (PB) of healthy donors, and of surgically excised adenoids of infants suffering of non-inflammatous hypertrophy by centrifugation of Ficoll-Hypaque gradient. CD14^+^ monocytes were isolated by the use of CD14-MicroBeads (Miltenyi Biotec,) and autoMACS-Pro isolation (Miltenyi Biotec). Maturation was done with heat-inactivated (h.i.) *C*. *albicans* (10 µg/ml, ATCC 10231) (Fig. S1), h.i. *A*. *fumigatus* (10 µg/ml, ATCC MYA-4609; protocol of Gaundar *et al*.^32^), Tetanus Toxoid (TT, 10 µg/ml) from *Clostridium tetani* (Calbiochem), staphylococcal enterotoxin B (SEB, 1 µg/ml) from *Staphylococcus aureus* (Sigma Aldrich), or fungal peptides PepMix™ Candida (MP65, 1 µg/ml) (JPT Peptides Technologies GmbH) over night at 37 °C in RPMI 1640 medium (Biochrom). The RPMI 1640 medium was supplemented with 10% Fetal Bovine Serum (Gibco/Life Technologies GmbH); 10 µg/ml streptomycin; and 10U/ml penicillin (Life Technologies GmbH). Monocytes were washed twice prior co-culturing with T cells.

**Figure 1:**
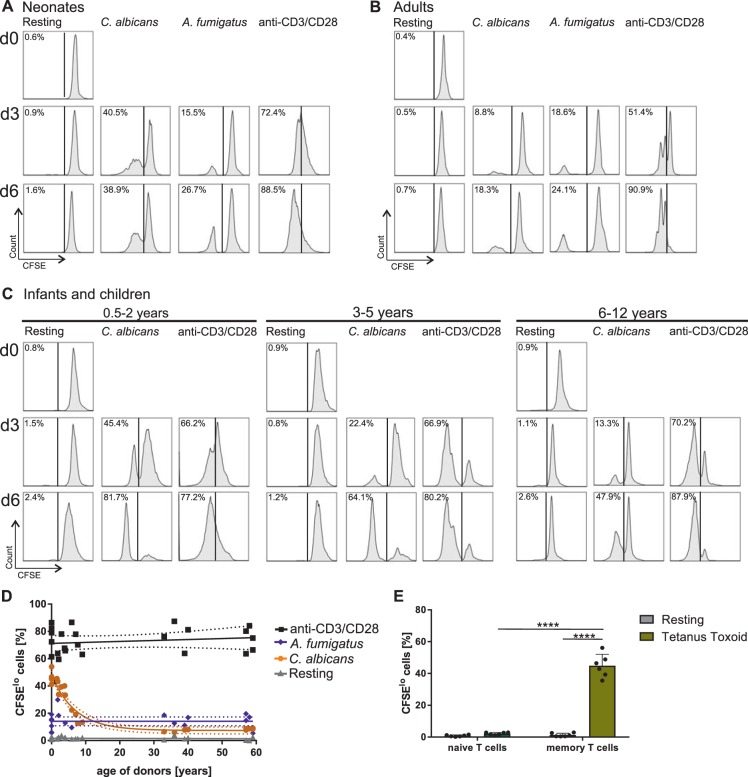
Fungi-specific T cell proliferation. (**A–C**) Purified CD4^+^CD45RA^+^ T cells were labelled with CFSE and cultured with monocytes matured with heat-inactivated *C*. *albicans* or *A*. *fumigatus* at a ratio of 2:1. CFSE dilution profiles and the frequency of proliferating (CFSE^lo^) T cells from neonates (**A**), infants and children (**C**) or adults (**B**) on day 3 and day 6 after stimulation. Data are representative of 5 donors. (**D**) Frequency of proliferating (CFSE^lo^) T cells from neonates, infants, children, and adults stimulated with *C. albicans* (orange), *A. fumigatus* (blue) or anti-CD3/CD28 (black) determined by flow cytometry are plotted against age. The dotted lines represent the 95% confidence interval. The coefficient of determination (R^2^) according to the one-phase decay exponential model in response to *C. albicans*-antigen is 0,9209. (**E**) Bar graphs showing frequency of naïve (CD4^+^CD45RA^+^CD31^+^) or memory (CD4^+^CD45RO^+^) T cells of children, expressing CD25 upon stimulation with Tetanus Toxoid for 3 days. Cumulative results are shown and each dot represents a different donor. The error bars in figures denote ± SD. ****p < 0.0001, as determined by one-way Anova with Tukey post hoc test.

**Figure 2:**
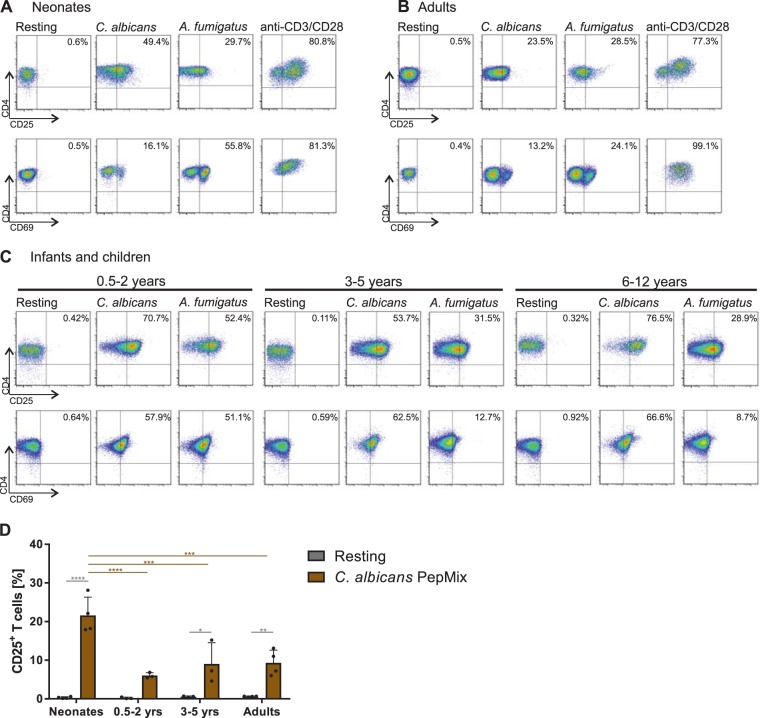
Fungi–specific up-regulation of activation markers. (**A–C**) Representative dot plots of flow cytometric analysis showing the frequency of CD4^+^CD45RA^+^ T cells of neonates (**A**), infants and children (**C**) or adults (**B**) expressing CD25 or CD69 after 3-day stimulation with monocytes matured with heat-inactivated *C*. *albicans* or *A*. *fumigatus*. Data are representative of at least 5 donors. (**D**) Bar graph showing CD25 expression of CD4^+^CD45RA^+^CD31^+^ T cells isolated form neonates, infants, children and adults in response to *C. albicans* PepMix (brown) as in (**A–C**). T cells were stimulated for 3 days and determined using flow cytometry. Cumulative results are shown and each dot represents a different donor. The error bars in figures denote ± SD. *p < 0.05, **p < 0.01, ***p < 0.001, ****p < 0.0001, as determined by one-way Anova with Tukey post hoc test.

**Figure 3:**
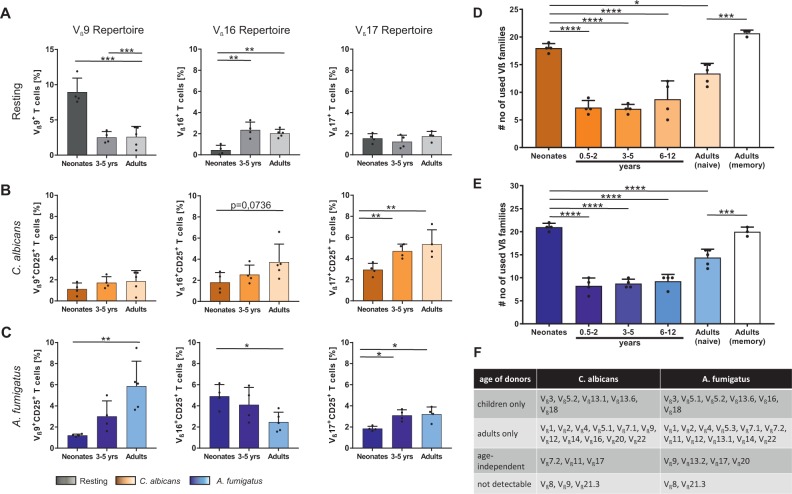
Fungi-induced T cell receptor V_ß_ family response. CD4^+^CD45RA^+^ T cells from neonates, infants, children, and adults were co-cultured with monocytes pulsed with *C*. *albicans-* or *A*. *fumigatus-*lysates and expression of different V_ß_ repertoire on T cells was measured by flow cytometry. (**A–C**) Frequency of V_ß_9 (left panel), V_ß_16 (middle panel) or V_ß_17 (right panel) expressing unstimulated T cells (**A**), T cells stimulated for 3 days either with *C*. *albicans* (B) or *A*. *fumigatus* (**C**) are shown. Each dot represents a different donor. (**D**,**E**) Bar graph showing TCR-V_ß_ expression either by CD4^+^CD45RA^+^CD25^+^ T cells from neonates, infants, children, and adults or by CD4^+^CD45RO^+^CD25^+^ T cells from adults. T cells are stimulated for 3 days either with *C*. *albicans* (orange) or *A*. *fumigatus* (blue). Cumulative results are shown and each dot represents a different donor. The error bars in figures denote ± SD. ^*^p < 0.05, ^**^p < 0.01, ^***^p < 0.001, ^****^p < 0.0001, as determined by one-way Anova with Tukey post hoc test (**A–C**) or Kruskal Wallis with Dunn’s post hoc test (**D** and **E**) (**F**) TCR-Vß repertoire usage in CD4 + CD45RA + T cells of infants and children as well as adults after co-culturing with monocytes pulsed with *C. albicans*- or *A. fumigatus*-lysates. The allocation of the groups took place if the expression of TCR-Vß family in the stimulated samples was higher than in unstimulated controls.

**Figure 4:**
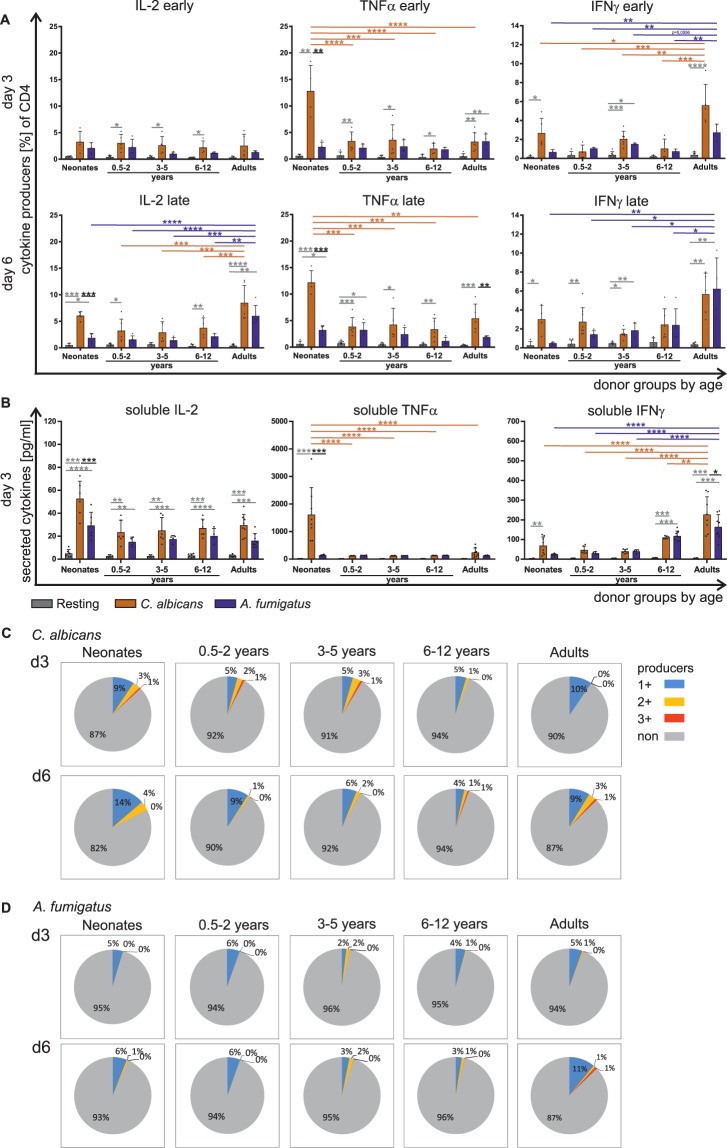
Fungi specific Th1 cytokine expression by T cells of different age groups. CD4^+^CD45RA^+^ T cells from neonates, infants, children, and adults were stimulated with *C*. *albicans* (orange) or *A*. *fumigatus* (blue) (as in Fig. 1) for 3 and 6 days respectively (**A**) Frequency of T cells expressing intracellular IL-2 (left panel), TNFα (middle panel) or IFNγ (right panel) was determined by flow cytometry. (**B**) Determination of IL-2 (left panel), TNFα (middle panel) or IFNγ (right panel) cytokine release of CD4^+^CD45RA^+^ T cells of neonates, infants and children or adults by LegendPlex which were either stimulated or not for 3 days. (**C**,**D**) CD4^+^CD45RA^+^ T cells were stimulated with *C*. *albicans* (**C**) or *A*. *fumigatus* (**D**) as in A, and the cells expressing single or multiple cytokines IL-2, TNFα, and IFNγ were determined by flow cytometry and analysed by Boolean gating and shown as fraction of all CD4^+^ T cells in a pie chart. The subsets that simultaneously express no (grey), one (blue), two (yellow) or three (red) different cytokines are grouped by colour. The data are representative of at least 5 donors. Cumulative results are shown and each dot in (**A**) and (**B**) represent a different donor. The error bars in figures denote ± SD. ^*^p < 0.05, **p < 0.01, ***p < 0.001, ****p < 0.0001, as determined by one-way Anova with Tukey post hoc test (**A**) or Kruskal Wallis with Dunn’s post hoc test (**B**).

**Figure 5:**
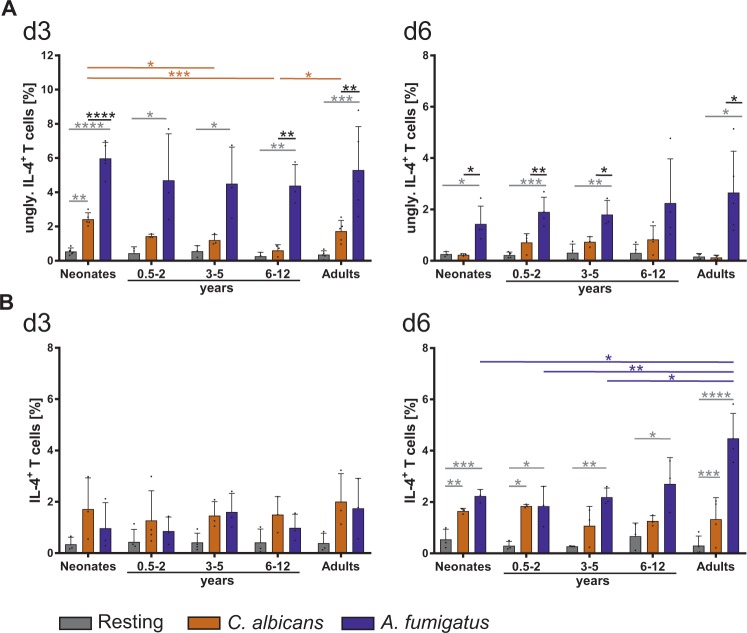
Age-dependent IL-4 production by fungi specific T cells. (**A**,**B**) CD4^+^CD45RA^+^ T cells from neonates, infants, children, and adults were stimulated with *C*. *albicans* (orange) or *A*. *fumigatus* (blue) (as in Fig. 4) for 3 (left panel) and 6 days (right panel) respectively, and analysed for the expression of intracellular un-glycosylated IL-4 isoform (upper panel) and mature IL-4 (lower panel). Cumulative results are shown and each dot represents a different donor. The error bars in figures denote ± SD. *p < 0.05, **p < 0.01, ***p < 0.001, ****p < 0.0001, as determined by one-way Anova with Tukey post hoc test.

**Figure 6:**
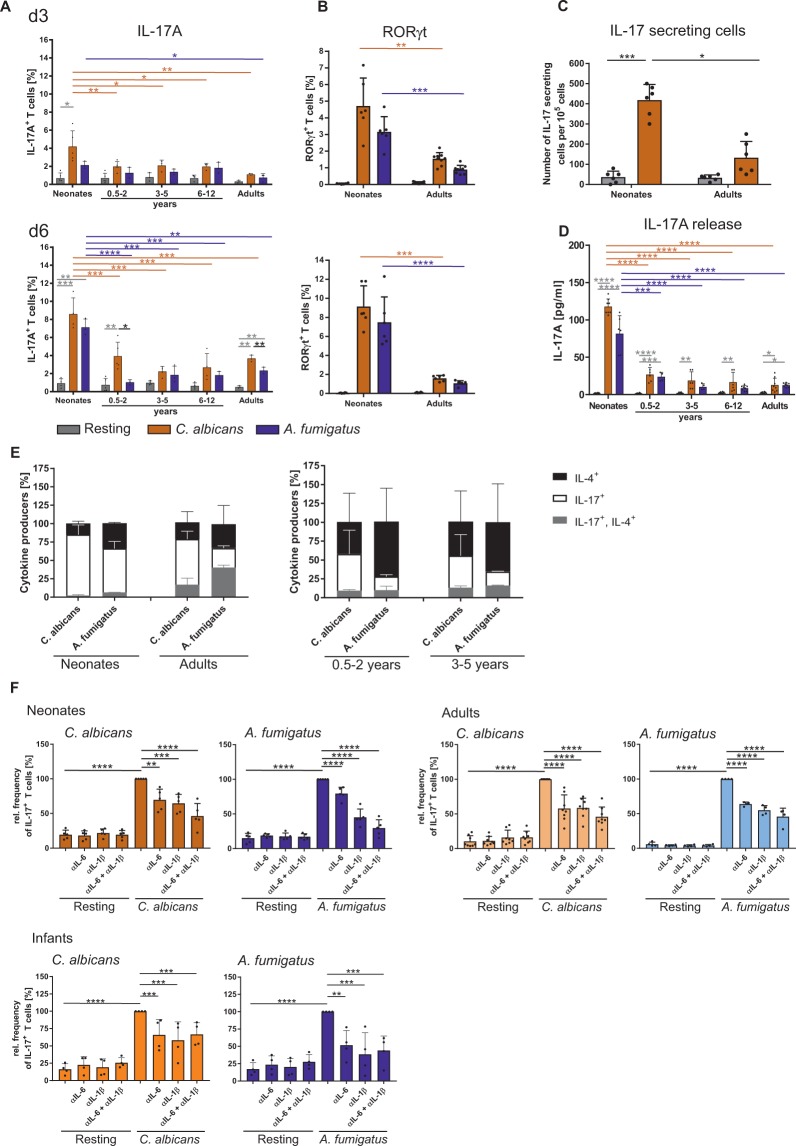
Fungi specific T cells produce IL-17 in an age dependent manner. CD4^+^CD45RA^+^CD31^+^ T cells from neonates, infants, children, and adults were co-cultured with monocytes pulsed with *C*. *albicans-* or *A*. *fumigatus-*lysates. (**A**,**B**) The frequency of T cells expressing signature Th17 molecules IL-17 (**A**) and RORγt (**B**) were analysed by flow cytometry at day 3 (upper Panel) and day 6 after stimulation (lower panel) (**C**) Bar graph representing the ELISPOT analysis of the quantitative IL-17, produced by the T cells from neonates and adults which were either stimulated or not for 3 days as described in (**A**). (**D**) Determination of IL-17A cytokine release of CD4^+^CD45RA^+^ T cells of neonates, infants and children or adults by LegendPlex which were either stimulated or not for 3 days as described in (**A**). (**E**) Frequency of T cells expressing intracellular IL-17 (white), IL-4 (black) and both IL-17/IL-4 (grey) were measured by flow cytometry after 6 days of stimulation, analysed by boolean gating and shown as different fractions of cytokine expressing cells in a stacked bar chart. **(F)** Bar graph showing IL-17 expression by CD4^+^CD45RA^+^CD31^+^ T cells of neonates, adults, and infants of 0.5-2 years old, stimulated for 6 days as described in (**A**) in the presence or absence of neutralizing antibodies for IL-1ß, IL-6 or both. Cumulative results are shown and each dot in (**A**–**D**) and (**F**) represent a different donor. The error bars in figures denote ± SD. *p < 0.05, **p < 0.01, ***p < 0.001, ****p < 0.0001, as determined by one-way Anova with Tukey post hoc test (**A-C, F**) or Kruskal Wallis with Dunn’s post hoc test (**D**).

CD4^+^CD45RA^+^ T cells or recent thymic emigrants (CD4^+^CD45RA^+^CD31^+^) were enriched to high purity (>98,5%) by magnetic beads separation with autoMACS-Pro using human naive CD4^+^ T Cell Isolation Kit or human CD4^+^ Recent Thymic Emigrant Isolation Kit (Miltenyi Biotec), respectively (Fig. S2). Only samples of >99,4% CCR7^+^ (Figs 1–5) or ^+^CD31^+^ T cells (Figs 6 and S8) of CD4^+^CD45RA^+^ T cells were considered naïve and used for cell assays. In 96-well plates, 5 × 10^5^/ml purified T cells were stimulated with the fungi-pulsed CD14^+^CD16^+^ non-classical monocytes (2.5 × 10^5^/ml purified monocytes) at a ratio 2:1 (T-cells/monocyte) for 3 or 6 days. For blockade of HLA-DR, monocytes were incubated with neutralizing anti-HLA-DR mAb (10 µg/ml, L249, purified from hybridoma, controlled by Western blotting and competitive FACS analysis), for 30 min at 37 °C in RPMI 1640 medium (Biochrom, supplemented as described above) prior to their maturation with antigens. Matured monocytes were washed twice, again incubated with anti-HLA-DR mAb for 30 min at 37 °C and co-cultured with T-cells as described above. Viability of monocytes upon anti-HLA-DR mAb treatment was controlled by manual gating of CD14^+^/CD16^+^/AnnexinV^−^/ propidium iodide^−^ cells (Data not shown).

Cytokines IL-6 and IL-1ß were neutralized using anti-IL-6 (10 µg/ml, MQ2-13A5, Biolegend) and anti-IL-1ß (10 µg/ml, 8516, R&D Systems) antibodies. For TLR inhibitor experiments, MyD88 inhibitor Pepinh-MYD (50 µM, InvivoGen) and anti-TLR2 (10 µg/ml, B4H2, InvivoGen) were used. For positive control, T cells were stimulated routinely with microbeads coated with anti-CD3 (1 µg/ml; UCHT1) and anti-CD28 (2 µg/ml; CD28.2) (both Biolegend) at a cell to bead ratio of 2:1.

### Flow cytometric analysis

Naïve (CD4^+^CD45RA^+^CD45RO^−^CCR7^+^CD25^−^CD8^−^) T cells were identified using specific fluorescent labelled antibodies (anti-CD4 (RPA-T4), anti-CD45RA (HI100), coupled anti-CD45RO (UCHL1), anti-CD25 (BL96); anti-CD8 (RTP-T8), and anti-CCR7 (GO43H7) (all Biolegend)) and isolated using FACS Aria (BD Bioscience) from the enriched CD4^+^CD45RA^+^ T cells. FcR Blocking Reagent (Miltenyi Biotec) was used to block Fc receptors according to the manufacturer’s instructions.

To determine cell proliferation, T cells were labelled with carboxyfluorescein succinimidyl ester (CFSE) (Molecular Probes/ Life Technologies GmbH) according to the standard protocol. CFSE-dilution at different time points was then analysed by flow cytometry.

Prior to intracellular cytokine analysis, naïve (CD4^+^CD45RA^+^CD8^−^CD25^−^) or recent thymic emigrant (CD4^+^CD45RA^+^ CD8^−^CD25^−^CD31^+^) T cells were pulsed with 10 ng/ml PMA, 1 µg/ml Ionomycin, and 5 µg/ml Brefeldin A (Sigma-Aldrich) for 4 h. Cells were fixed with 2% paraformaldehyde (Morphisto GmbH) in PBS for 20 min, following permeabilized with 0.5% saponin (Sigma-Aldrich) in PBS/BSA, and then incubated with the following antibodies: anti-IL-17A (eBio64DEC17, eBioscience), anti-IFNγ (4 S.B3; BD Biosciences), anti-CD4 (RPA-T4), anti-CD3 (SK7); anti-CD25 (M-A251), anti-CD45 (HI30), anti-IL-2 (MQ1-17H12), anti-IL-4 (MP4-25D2, for detection of mature IL-4), anti-IL-4 (8D4-8; for detection of IL-4 isoform) and anti-TNFα (Mab11) (all Biolegend, San Diego, USA). To analyse activation-induced surface molecules by flow cytometry, cells were harvested and stained with anti-CD4 (RPA-T4), anti-CD3 (SK7); anti-CD45 (HI30), anti-CD25 (M-A251), and anti-CD69 (FN50) (all Biolegend) in PBS/BSA.

To measure transcription factors by flow cytometry, cells were fixed with 4% formaldehyde (Carl Roth) in PBS for 10 min at 37 °C followed by permeabilization in ice cold 90% methanol (Carl Roth) for 30 min, and then stained with antibodies for specific transcription factors anti-RORγt (eBioscience) and anti-T-bet (Biolegend).

For the assessment of TCR-V_ß_ expression we used IOTest Beta Mark TCR-V_ß_ Repertoire kit (Beckman Coulter) for quantitative analysis of the most abundant 24 TCR-V_ß_ clonotypes. To identify activated T cells, cells were co-stained with anti-CD4 (RPA-T4), anti-CD3 (SK7); anti-CD45 (HI30), anti-CD69 (FN50), and anti-CD25 (M-A251) (all Biolegend). All flow cytometric analyses were performed using FACS Canto II (BD Biosciences) together with FACSDiva software (BD Biosciences) to collect and compensate the data and FlowJo software (FlowJo LLC) for final data analysis.

### EliSpot assays

ELISPOT plates (Millipore 96-well MultiScreen HA; Millipore) were coated with 2 mg/ml anti-human IL-17–specific mAb (eBio64CAP17; eBioscience) in PBS. Cells were cultured in the presence of PMA and ionomycin for 18 h at 37 °C/5% CO_2_ and plated at a density 1,25 × 10^5^ cells/well in the ELISPOT plate. Cytokines IL-17 and IFNγ were detected with biotinylated anti-human IL-17 or IFNγ Abs, respectively (eBio64DEC17; eBioscience) and developed using 0.3 mg/ml extravidin-alkaline phosphatase (Sigma-Aldrich) and an Alkaline Phosphatase-substrate Kit III Vector Blue (Vector Laboratories). Spots were counted with ImageJ.

### Analysis of cytokines in T cell culture supernatant

Levels of IFNγ, TNFα, IL-2, and IL-17A, in human T cell assay supernatants were measured using LEGENDplex human Th Cytokine Panel (Biolegend) according to the manufacturer’s instructions. Samples were diluted 1:3 in assay buffer. Analyses were performed using FACSCanto (Becton Dickinson) and LEGENDplex analysis software (VigeneTech Inc.).

### Statistical analyses

Statistical analyses and cumulative data presentation were performed with Prism 7 (GraphPad 7 software Inc.). We used Shapiro-Wilk test for testing of normality. Comparison of distribution was performed using two-tailed Student’s t-test or Wilcoxon test for comparison of two parameters; one-way-ANOVA or Kruskal-Wallis test for multiple comparisons depending on the results of Shapiro-Wilk test, with P < 0.05 (*), P < 0.01 (**) and P < 0.001 (***) indicating statistically significant differences.

## Results

### T cells of neonates and infants respond to C. albicans and A. fumigatus extensively

As neonatal and infant T cells are thought to be hyporesponsive, we investigated whether naïve CD4^+^ T cells from neonates and infants are able to respond efficiently to *C*. *albicans* and *A*. *fumigatus* antigens presented by autologous APCs (Fig. S1), respectively. Isolation of CD4^+^CD45RA^+^ T cells from neonates, infants, children, and adults which had no history of recurrent infections or inflammations revealed routinely >99,4% CD31^+^ cells (adults >98,6%) and showed similar results as FACS sorted CD4^+^CD45RA^+^CCR7^+^ T cells (data not shown, Fig. S2), and were therefore considered having a naïve phenotype (Figs 1–5). Therefore, we isolated CD4^+^CD45RA^+^ T cells with a purity of routinely >99,6% CD31^+^ from cord blood and used them as a specimen of “youngest” T cells in comparison to peripheral blood of healthy adult donors. T cells were labelled with carboxyfluorescein succinimidyl ester (CFSE) and stimulated with *Candida*- or *Aspergillus* –loaded monocytes, respectively (Fig. 1). In comparison to T cells from adults, T cells from neonates displayed a higher frequency of proliferation (CFSE^low^) after stimulation with *C*. *albicans* for a period of 3 and 6 days (Fig. 1A). When comparing the CFSE dilution profiles, however, it is noticeable that higher frequencies of neonatal T cells undergo cell division although by dividing less frequently. Indeed, calculating the precursor frequency demonstrates that 10 times more neonatal than adult T-cell precursors respond to *C*. *albicans* (10.248% ± 1.508 versus 1.226% ± 0.263). Nevertheless, frequency of proliferating cells in response to *A*. *fumigatus* was observed to be similar in neonatal and adult T cells, as is the precursor frequency.

To address whether T cells from infants and children, also exhibit enhanced proliferative capacity against *C*. *albicans* antigens, we enriched CD4^+^CD45RA^+^ T cells routinely from adenoids of infants and children that were eventually >99,4% CD31^+^ of the CD4^+^CD45RA^+^ T cells (Fig. S2) and stimulated them with APCs pulsed with *C*. *albicans* antigens (Fig. S1). The frequency of T cell proliferation in response to *C*. *albicans* was determined and showed higher frequencies of proliferating T cells of 45% in the youngest donors of 0.5-2 year olds, which then decreased with age to 22% in 3–5 year olds followed by 13% in 6–12 year olds (d3, Fig. 1C). Similar results were obtained investigating activation-associated molecules on T cells (Fig. 2A-C). Proliferative responses of the T cells mediated by polyclonal antigen-independent stimulation using anti-CD3 plus anti-CD28 crosslinking were used as a control to compare the efficiency of T cell proliferation in response to *C*. *albicans* (Fig. 1A,B, right). Combined data of all donors examined revealed a strong correlation with age in response to *C*. *albicans* (R^2^ = 0,9209) showing highest frequencies of proliferating T cells from neonates and infants up to 5 years of age (Fig. 1D). Thereafter decrease continued being lowest for adults. In comparison, *A*. *fumigatus* induces a similar amount of T cell proliferation in neonates as in children and adults (Fig. 1D). In respect to antigen-specificity, proliferation of T cells from all ages was significantly reduced upon blockade of HLA-DR, respectively (Fig. S3). Furthermore, we have stimulated naive T cells with tetanus toxoid peptides to unambiguously assess the quality of isolated naive T cells (Fig. 1E). In accordance with other studies^33^, only memory T cells - even decades after vaccination - proliferate in response to TT, but not naive ones.

In addition, to underpin the antigen specific responsiveness towards *C*. *albicans*, we stimulated CD4^+^CD45RA^+^CD31^+^ T cells 3 days with monocytes matured with *C*. *albicans*-antigens or with a commercial *C*. *albicans* peptide mix consisting of 92 peptides of *C*. *albicans*, respectively (Fig. 2D). To more clearly visualize the antigen-specificity and the age-dependent effect of *C*. *albicans*, a synthetically generated peptide mix of *C*. *albicans* proteins was used. In line with our results using fungal lysates (Fig. 2A) results from C. albicans peptide mix show that CD4^+^CD45RA^+^CD31^+^ T cells from neonates are activated much more frequently than from children and adults (Fig. 2D). Both antigenic stimuli unambiguously induced CD25 and CD69 expressing cells in comparison to unstimulated conditions. Although fungus-pulsed APCs were washed prior contact with T cells, T cells were pre-incubated with the inhibitor of TLR signalling molecule MyD88 or blockade of TLR2 with specific antibodies and showed no effect on stimulation (Fig. S4). Activity of TLR inhibitor in these experiments was controlled on SEB-activated monocytes (Fig. S5). These results indicate that the compartment of naïve T cells of neonates and infants is ready to mount vastly a response against fungal-antigens presented on autologous APCs and - at least for *C*. *albicans* - even stronger than stimulated naive T cells of older children or of the adult naive T-cell compartment.

### Pre-existing T cell repertoires of fungus reactive T cells

To investigate the TCR diversity of the responding cells, we isolated naïve T cells to a purity of >99,4% CD31^+^ of CD4^+^CD45RA^+^ T cells from infant donors of different ages and determined the quantitative and qualitative expression of TCR-V_ß_ subpopulations against *C*. *albicans* and *A*. *fumigatus* (IOTest Beta Mark TCR-V_ß_ repertoire kit). Within 3-days of fungus-specific stimulation, we found that activated CD25^+^CD4^+^ T cells showed a non-random usage of the TCR-V_ß_ repertoire (Fig. 3). Interestingly, highest number of different V_β_ subfamilies was used by CD25^+^CD4^+^ T cells from neonates (Fig. 3D,E). However, a dramatic reduction in TCR-V_ß_ usage against fungi was observed between T cells from neonates and those of small infants aged 0.5–2 years. Subsequently, number of V_β_ subfamilies used against fungi increased steadily with age from infants to adults. Nevertheless, many different V_β_ families were uniquely used for the antifungal T-cell response of neonates. Based upon these results, we examined the possibility that V_β_ subfamilies missing in the naïve compartment might reappear in the memory compartment of adults. Compared with the naïve compartment, the number of V_β_ families used in memory pool is significantly increased (Fig. 3D,E). The TCR frequency from donors of variant age groups that use 3 different V_ß_ families (V_ß_9, V_ß_16, V_ß_17) are plotted as examples in Fig. 3A–C. TCR distribution appears to be varying in resting cord blood T cells with low frequencies of T cells (0.45 ± 0.45%) expressing V_ß_16 and a high frequency of them (8.9 ± 1.9%) expressing V_ß_9. Intriguingly this pattern of TCR distribution was not observed after T cell stimulation, as antigen-specific stimulation of neonatal T cells with *C*. *albicans-* or *A*. *fumigatus*-pulsed monocytes led to an increased usage of V_ß_16 and V_ß_17 subfamily and a decreased usage of V_ß_9 subfamily (Fig. 3A–C), respectively. The expression pattern of TCR-V_ß_ subfamilies in infants and children show a predominant usage of V_ß_3, V_ß_5.2, V_ß_13.6 and V_ß_18 in reply to fungal-antigens that differs from adults. Of note, activated T-cells expressing V_ß_8 and V_ß_21.3 were not detectable in response to fungal antigens (Fig. 3F). However, many V_β_ families were uniquely used in the antifungal T-cell response of neonates and infants. These data show that the antigen-specific T cell responses to *C*. *albicans-* and *A*. *fumigatus*-pulsed monocytes use a broad repertoire implying that T cells of neonates and young children are predetermined to expand to fight fungal pathogens.

### *C. albicans* and *A. fumigatus* mediateTh1 cell differentiation in an age dependent manner

To decipher the age-dependent diversity of fungi-specific T cells in detail, we first analysed expression and accumulation in the supernatants of Th1-like cytokines, namely IL-2, TNFα and IFNγ. CD4^+^CD45RA^+^ T cells from representative donors of different ages in response to *C*. *albicans-* and *A*. *fumigatus*-pulsed monocytes are shown in Fig. 4A,B. Fungus-specific production of Th1-associated cytokines IL-2 and IFNγ was identified in T cells of neonates, infants, and children to be low (Figs 4A,B and S6). Representative flow plots with CD4 and cytokine co-expression are depicted in S7. However, 3–4 times more TNFα producing T cells were observed in response to *C*. *albicans* in neonatal samples compared to T cells of infants or adults after stimulation for 3 and 6 days, whereas all donors examined responded similarly to A. *fumigatus* in inducing TNFα producers (2–3%). No difference could be detected between enriched CD4^+^CD45RA^+^ and CD4^+^CD45RA^+^CD31^+^ T cells at any age group in frequency of cytokine responders to *C*. *albicans* or *A*. *fumigatus* (Fig. S6). To ensure that difference in responding T cells indeed translates into differences of secreted TNFα, it was analysed in supernatants from 4 donors of each of the 5 age-groups (Fig. 4B). Indeed, significantly enhanced concentrations of TNFα were monitored in samples of neonates.

By analysing flow cytometric data with Boolean gating, multifunctional T cells were characterized to determine functionality of the T-cell response following stimulation with *C*. *albicans-* or *A*. *fumigatus*-antigen-matured monocytes. Although Th1-like cells were observed to be developed at any age, analysis of multi-functional CD4^+^CD45RA^+^ T cells showed that T cells isolated from neonates, infants, and children up to 5 years of age developed double and triple producers already after 3 days of stimulation with *C*. *albicans*, while T cells from adults did so only at a later stage (Fig. 4C). In addition, significantly different frequencies of cytokine producers were initiated by each fungus; of note, single producers were mainly induced by both of them in T cells from neonates and children up to 2 years of age (Fig. 4D).

### Th-2-like cytokine production is initiated in response to *A. fumigatus*

As infants are discussed to have a bias for Th2-like responses^24^, we investigated the frequency of Th2-like cells expressing cytokines IL-4 and IL-13 in response to fungi (Fig. 5, S9). In general, the frequencies of T cells responding against *A*. *fumigatus* with unglycosylated IL-4 production were 2–4 times higher than those against *C*. *albicans* across all age groups. Interestingly, a frequency of 5% unglycosylated IL-4 expressing T cells are detectable after 3 days of stimulation in all age groups implying that the IL-4 machinery is indeed activated in response to *A*. *fumigatus* independently of age (Fig. 5A). Mature IL-4 producers were detectable upon 6 days after beginning of the stimulation with *A*. *fumigatus* in all age groups, with adults displaying a frequency a mature IL-4 producers as high as glycosylated IL-4 producers after 3 days of stimulation (Fig. 5B). At day 6, *C*. *albicans*-specific responses also contained IL-4 producers (2%), but with no significant age differences. Of note, IL-13 expression is not significantly induced (Fig. S9). However, taken together, Th2 machinery is switched on in response to fungi at any age.

### Neonates respond to *C. albicans* and *A. fumigatus* with IL-17 producing T cells

For studying age-dependent peculiarities in IL-17 production, we isolated CD4^+^CD45RA^+^CD31^+^ T cells and stimulated them with *C*. *albicans-* or *A*. *fumigatus*-pulsed monocytes. The quantitative analysis of *C*. *albicans*-specific cytokine expressing cells by flow cytometry, soluble cytokine in supernatants and cytokine secretion by EliSpot assay, displayed highest frequencies of IL-17^+^ fungus-specific T cells in neonatal samples compared to those from infants or from adults (Fig. 6A,C,D). Also the frequency of cells expressing IL-17 transcription factor RORγt is 2 times more in T cells from neonates than in T cells from adults (Fig. 6B). Next, co-expression of IL-4 with IL-17 was monitored showing higher frequencies in adults after stimulation with *A*. *fumigatus*, than from neonates or children up to 5 years of age (Fig. 6E).

To further investigate whether the enhanced induction of IL-17 by neonatal T cells upon stimulation with fungi is dependent on IL-1ß and IL-6, we isolated CD4^+^CD45RA^+^CD31^+^ naïve T cells from donors of different ages as indicated and stimulated them with specific fungus in the presence or absence of blocking antibodies against IL-1ß and IL-6. As shown in Fig. 6F, neutralization of IL-1ß and IL-6 by specific antibodies significantly reduced the generation of IL-17 producers by both fungi in all age groups.

**Figure 7:**
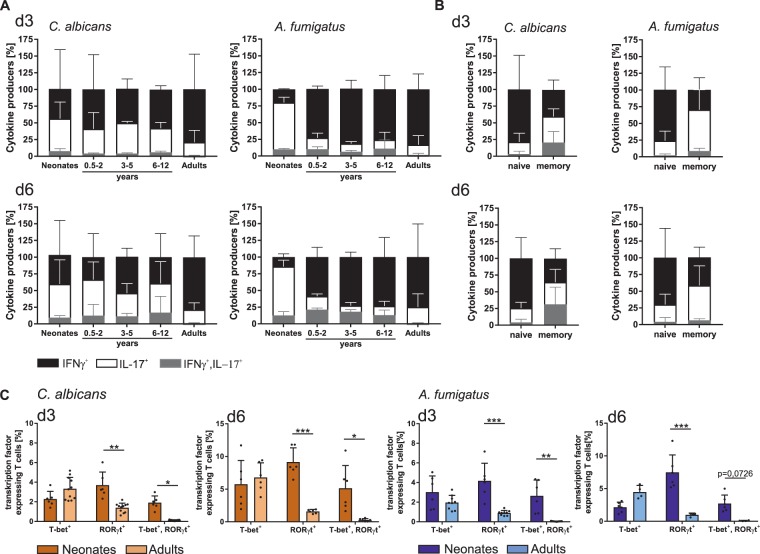
Identification of age-dependent fungi specific multifunctional Th1/Th17 cells. (**A**) CD4^+^CD45RA^+^CD31^+^ T cells from neonates, infants and children of different age groups, and adults were co-cultured with monocytes pulsed with *C*. *albicans* (left panel) or *A*. *fumigatus* (right panel). The frequency of T cells expressing intracellular IL-17 (white), IFNγ (black) and both IL-17/IFNγ (grey) were determined by flow cytometry at the indicated time points, analysed by boolean gating and shown as different fractions of cytokine expressing cells in a stacked bar chart. (**B**) Frequency of naïve (CD4^+^CD45RA^+^CD31^+^) or memory (CD4^+^CD45RO^+^) T cells of adults expressing intracellular IL-17 (white), IFNγ (black) and both IL-17/IFNγ (grey) were determined by flow cytometry at the indicated time points and analysed as described in (**A**). (**C**) Frequency of CD4^+^CD45RA^+^CD31^+^ T cells from neonates and adults expressing Th1 and Th17 transcription factors T-bet, RORγt and both T-bet as well as RORγt following stimulation with *C*. *albicans* (orange bars) or *A*. *fumigatus* (blue bars) for 3 and 6 days as described in (**A**). Cumulative results are shown and each dot represents a different donor. The error bars in figures denote ± SD. *p < 0.05, **p < 0.01, ***p < 0.001, ****p < 0.0001, as determined by Kruskal Wallis test with Dunn’s post hoc test.

Next, we investigated the capacity of *C*. *albicans*- and *A*. *fumigatus*-primed IL-17 expressing T cells to co-produce IFNγ. Upon fungus-specific stimulation, T cells from neonates, infants, and children displayed an ability to co-produce cytokines IL-17 and IFNγ (Fig. 7A), which was hardly detectable in responding naïve T cells from adults. Co-producing T cells appeared indeed only in the memory compartment of adults (Fig. 7B). As co-production of these 2 cytokines would require transcription factors RORγt and T-bet to be co-expressed in individual cells, the expression of these transcription factors were deciphered in stimulated CD4^+^CD45RA^+^CD31^+^ T cells by FACS analysis. Indeed, a high frequency of T cells from neonates but not adults unambiguously co-expressed RORγt and T-bet (Fig. 7C). These results demonstrate that early in life, a high plasticity of Th subsets exists.

## Discussion

In this study, we investigated the antigen-specific cellular and molecular mechanisms of differentiation of the naïve CD4^+^ T cells from neonates, infants, children, and adults in response to *C*. *albicans* and *A*. *fumigatus*. Our data revealed the novel observation that unlike T cells from adults, especially in early life, a broad repertoire of antifungal naïve CD4^+^ T cells respond with vast proliferation. Responding T cells showed high frequencies of IL-17 producers and co-expression of transcription factors of different Th lineages. Th cell differentiation against fungi was indeed age-dependent with signatory elements; however, the anti-fungal T cell responses were strongly determined by each individual pathogen.

Within fungus-activated T cells, we found a non-random usage of TCR V_ß_ repertoire. To our knowledge, we are the first to show the TCR V_ß_ repertoire in response to *C*. *albicans-* and *A*. *fumigatus-*antigens. As the T cell response is fast and extraordinarily high for neonates, a predetermined repertoire with inherited fungus-specific regions seem to be likely, which also have been recently described for malaria-antigens and a respiratory syncytial virus fusion glycoprotein^34,35^. This brings us back to the main function of the immune system: fighting pathogens and it might be likely that even a more predetermined repertoire against pathogens will be identified in the near future. The expression pattern of TCR V_ß_ subfamilies in infants and children show a predominant usage of V_ß_3, V_ß_5.2, V_ß_13.6 and V_ß_18 in reply to fungal-antigens which differs a lot from adults. In terms of T cell repertoire of resting CD4^+^CD45RA^+^ T cells from donors of different ages, our analysis confirms and extends previous results from total CD4^+^ T cells in whole blood^36–38^, besides a lower frequency of V_ß_2 chain but a higher frequency of V_ß_9 chain in isolated neonatal naive T cells compared to total CD4^+^ T cells^36^. However, the relative TCR V_ß_ repertoire of resting T cells does not reflect the T cell repertoire pattern upon fungus-specific activation. It is known from literature that some of the most significant TCR clusters in different human donors seem to be associated with common viral pathogens such as parvovirus b19, influenca, CMV and Epstein Barr virus^39^. Beyond that, *Segmented Filamentous Bacteria*, members of the gut microbiota of rodents, fish and chickens, induces in mice a strong Th17 response characterized by an enrichment of V_ß_14 TCR within the Th17 cells. These V_ß_14 TCR Th17 cells do not respond to *Listeria monocytogenes*, a strong inducer of Th1 cells^40^. In addition, it was also shown that a naïve T cell population expressing a fixed TCR repertoire occurs in response to commensal bacteria in colonic regulatory T cell population but not in effector T cell pool^41–43^. Together with these previous studies, our findings strongly suggest that a particular repertoire exists for certain antigen specificity.

In case of fungi provocation, Th17 cells especially play an important role in defending the organism^44^. Our data show that CD4^+^CD45RA^+^CD31^+^ T cells from neonates display a particularly high signatory IL-17 response to *C*. *albicans* in a fast manner in comparison to *A*. *fumigatus*. A signatory IL-17 production by T cells from neonates upon unspecific, polyclonal stimulation has also been reported^45^. Therefore, it is likely that the quick IL-17 response especially against *C*. *albicans* at birth is predetermined by a pronounced usage of TCR V_ß_. In terms of co-evolution of *Candida A*. and the human immune system, it is likely that neonates are confronted with *Candida* – at least – during birth through vertical transmission from the mother’s microbiome, thus, it is life-saving for neonates to be able to initiate anti-*Candida* reactions quickly. Thereafter in life, IL-17 response is decreased against *C*. *albicans* and almost absent against *A*. *fumigatus*. Recently, in samples from adult donors an expansion of Treg cells rather than naïve T cells in response to *A*. *fumigatus* was described^46^. *Aspergillus*-specific T cells from PBMCs of adult healthy individuals were shown to have a predominant Th1-like phenotype, whereas *C*. *albicans*-reactive T cells have been shown to produce mainly IL-17^29^. According to our data, these cells likely are reactivated from the effector/memory pool, as in our hands naïve adult T cells were not able to produce substantial amounts of IL-17. Indeed, we could detect some of these cells within the fungi-specific stimulated memory/effector pool (Fig. 7B). High amounts of *Aspergillus*-specific IL-17-expressing T cells so far could only be observed in lung of adult COPD patients^18^. We have also observed *Aspergillus*-specific IL-17-expressing T cells from adults in our hands, but these cells also displayed a strong Th2-like phenotype at the same time. Thus, it would be interesting to see whether the IL-17 expressing cells from COPD patients indeed co-express IL-4, especially in response to *A*. *fumigatus* as it was reported for patients with severe asthma^47^ and this beneficial information could open doors to design future therapies.

Our results demonstrate that fungus-matured monocytes from neonates as well as from children and adults are potent APCs (Fig. S1);^48,49^ monocytes show potent cytokine production and CD14 and CD16-coexpression in response to fungal antigens indicating that monocyte responses to fungi at birth and early in life are not deficient^50,51^. Whereas we cannot detect significant difference of fungus-matured monocytes between ages (Fig. S1B), others reported contradicting results^52,53^. For example, a lower capacity of umbilical cord blood monocytes to produce IL-1β and TNFα in response to Tuberculin Purified Protein Derivative (PDD) was reported to be due to a functional immaturity of cord blood monocytes at the cellular level^54^. Seemingly at odds with these results, previous reports have also demonstrated that monocytes from cord blood express similar or higher levels of TNFα, IL-6 and IL-1ß in response to peptidoglycan or TLR agonist panel^50,55,56^. At first sight discrepancies could be due that suboptimal or artificial provocations of the monocytes were applied and could indeed reveal age-related differences^50,54–56^. Similar maturation of monocytes in our setting might be due to applying physiological proteins of pathogens in excess, which likely equals out age-related differences. Appealing from an evolutionary point of view, this would ensure that monocytes at any age are able to become potent APCs in response to fungal threat.

In line with the previous findings that IL-6 and IL-1ß produced by APCs, are important for inducing IL-17 production by CD4^+^ T cells^9,10^, we have observed that blockade of IL-6 or IL-1ß or both reduced IL-17 producers in fungus-specific CD4^+^CD45RA^+^CD31^+^ T cells from all age groups. The importance of IL-1β secretion during *C*. *albicans* infection is highlighted by the findings that mice deficient in IL-1β receptor are highly susceptible to disseminated candidiasis^57^. However, an approximately 50% total Th17 producers were IL-6 and IL-1ß independent, indicating that the residual Th17 cells could be originated intrathymically (nTh17 cells). Alternatively IL-23 might also take over the differentiation of Th17 cells^58^. Stimulation of CD4^+^CD45RA^+^CD31^+^ T cells with fungi-pulsed monocytes also displayed a co-expression of cytokines IL-17 and IFNγ simultaneously, with higher frequencies of these cells detected in neonates and infants. Others have shown that Th1/Th17 cells were also detected at low levels in *C*. *albicans* stimulated adult PBMCs^59^, however our data implicate that these cells likely arise from the memory pool (Fig. 7A). In accordance with the cytokine production profile, the transcription factor analysis also clearly demonstrated that CD4^+^CD45RA^+^CD31^+^ T cells from neonates expressed high levels of RORyt and T-bet simultaneously in response to fungal-antigens. These differences in Th1/17 subpopulation likely points towards age-related differences in plasticity of T cell differentiation. These results are in line with studies showing that naive CD4^+^ T cells generate precursors that are still multipotent^10^ and that they are able to produce a heterogeneous progeny, as well as plasticity of T cells in the human immune response^60^. More precise knowledge of this plasticity of Th cells from neonates and infants could open new avenues for therapy aiming for (re)programming the most optimal response.

Taken together, our study provides a better understanding of age-related immune responses against *C*. *albicans* and *A*. *fumigatus*, two harmful human pathogens. Here, we show that subpopulations of CD4^+^ T cells especially from neonates and infants respond extensively from birth on to *C*. *albicans* and *A*. *fumigatus* without a bias for Th2. Although cytokines were produced at any age, T cells of neonates show a signatory IL-17 response to fungi accompanied by IL-17/IFNγ co-expressing T cells. Together with rapid generation of multifunctional anti-fungal T cells, the adaptive immune system of neonates and young children are already well equipped to fight fungal pathogens. The unique response against specific fungi and the relation to the age of the affected organism might pave the way to more specific interventions with less devastating side effects than currently used drugs.

## Electronic supplementary material

Supplemental Figures
