# Supplementary material for: Developmental induction of human T-cell responses against Candida albicans and Aspergillus fumigatus
Source: Sci Rep. 2018 Nov 15;8:16904. doi: 10.1038/s41598-018-35161-5 (PMC6238003; doi:10.1038/s41598-018-35161-5)
Supplement: Supplementary file 1 — Supplemental Figures [file 41598_2018_35161_MOESM1_ESM.pdf]

Supplemental Figures

Developmental induction of human T-cell responses against *Candida albicans* and *Aspergillus fumigatus*

Katrin Vogel, Mandy Pierau, Aditya Arra, Karen Lampe, Dirk Schlueter, Christoph Arens, and Monika C. Brunner-Weinzierl

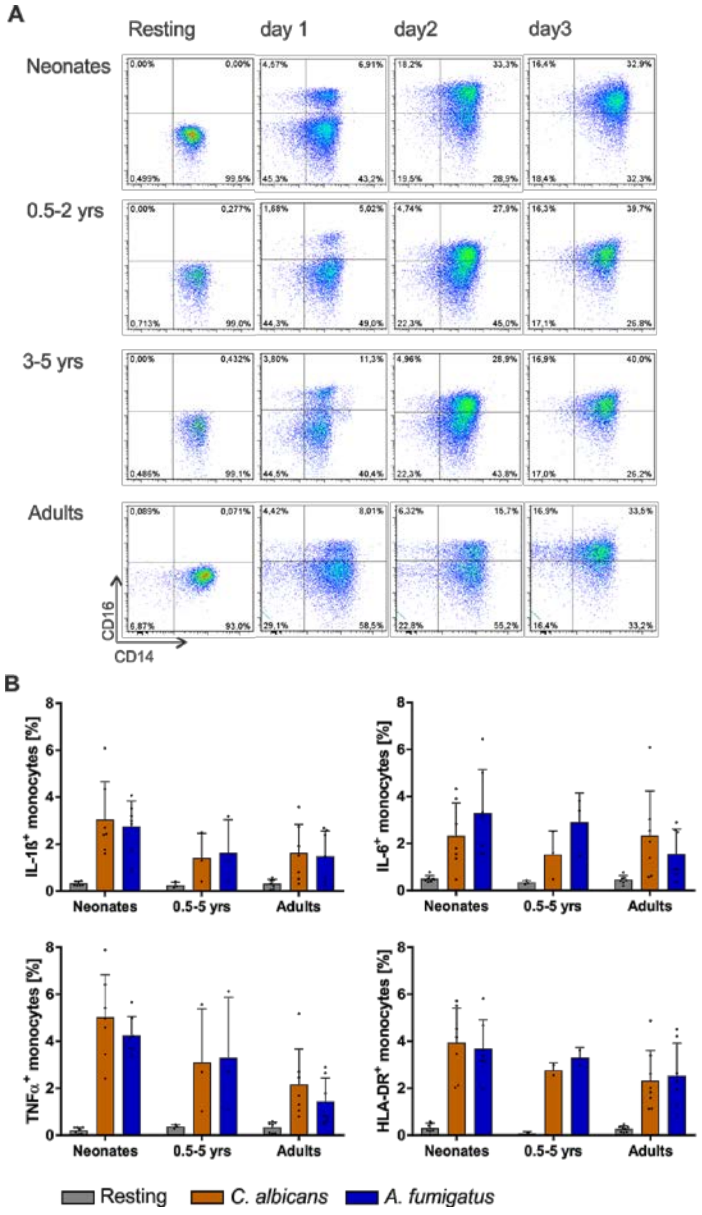

Figure S1. **Maturation of isolated monocytes**

(**A**) CD14+ monocytes from neonates, infants and children of different age groups, and adults were cultured with *C. albicans* for the indicated time points. Expression of surface molecules CD16 and CD14 were measured by flow cytometry. The data are from a single donor and is a representative of 3 donors. (**B**) Bar graphs showing the frequency of CD14+ monocytes from neonates, children, and adults (after simulation with *C. albicans* (orange bars) or *A. fumigatus* (blue bars) for 24 hours) expressing the cytokines IL-8, IL-6, IL-1ß, and TNFα as well as the surface molecule HLA-DR. Cumulative results are shown and each dot represents a different donor. The error bars in figures denote ± SD. Age-specific cytokine expression were not significantly different as determined by Kruskal Wallis test with Dunn's post hoc test.

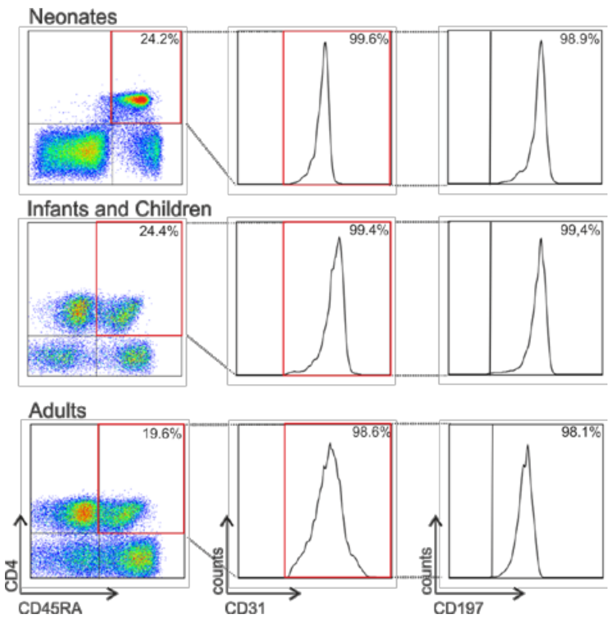

Figure S2. **Frequencies of T cell subsets**

Flow cytometric dot plots and histograms are shown with frequency of $CD4^{+}CD45RA^{+}$ T cell subsets from neonates (top), infants and children (middle, 2 years) and adult (bottom) samples. The data are from a single donor each and is a representative of 5 donors per age group.

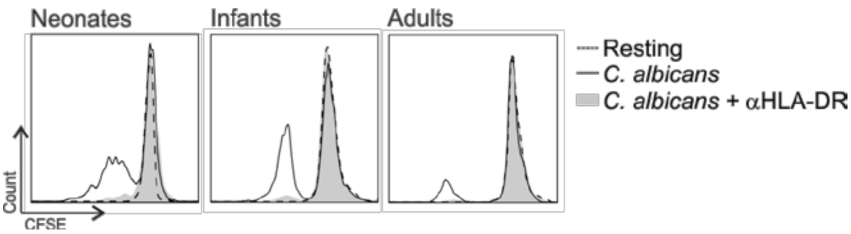

Figure S3. **Antigen specificity of T-cell proliferation**

To investigate antigen specificity of T cell proliferation, monocytes pre-treated with heat-inactivated *C. albicans* in the presence or absence of anti-human HLA-DR antibody were co-cultured with CFSE labelled $CD4^+CD45RA^+$ T cells from neonates (left), infants and children (middle, 2 years), and adults (right). 3 days later frequency of proliferating ($CFSE^{lo}$) T cells was determined using flow cytometry. Data are representative of 5 donors.

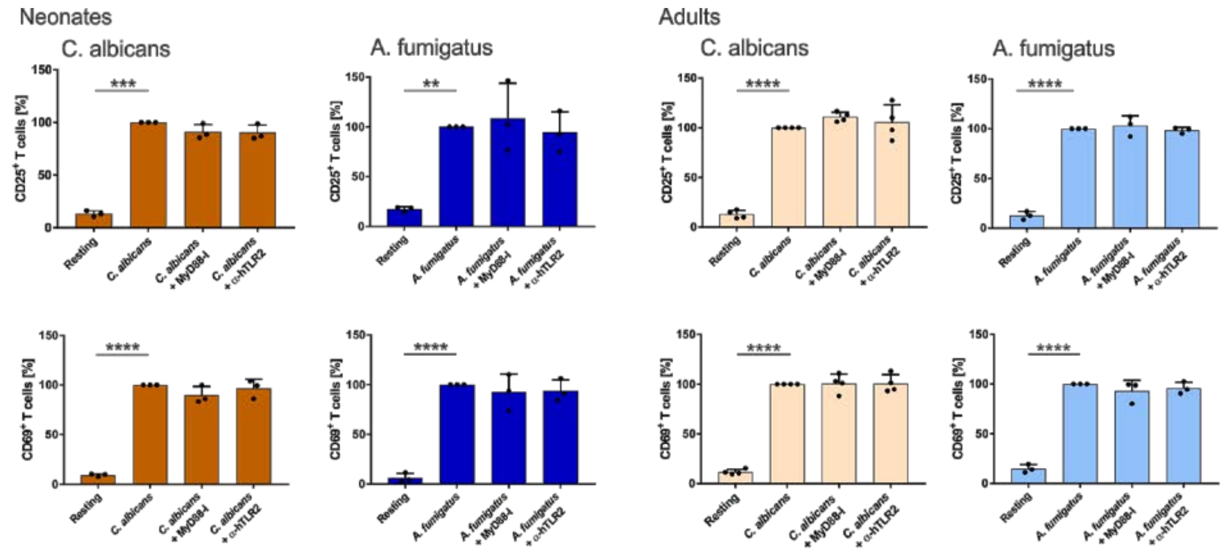

<u>Figure S4.</u> **T-cell activation is TLR-independent**

$CD4^+CD45RA^+CD31^+$ T cells from neonates and adults were co-cultured with monocytes pulsed with *C. albicans*- or *A. fumigatus*-lysates in the presence or absence of MyD88-inhibitor or anti-human TLR-2 antibody. Surface expression of CD25 and CD69 was measured by flow cytometry and the frequency of these cells from neonates and adults are presented as bar graphs. Cumulative results are shown and each dot represents a different donor. The error bars in figures denote ± SD. **$p<0.01$, ****$p<0.0001$ as determined by one-way Anova with Tukey post hoc test.

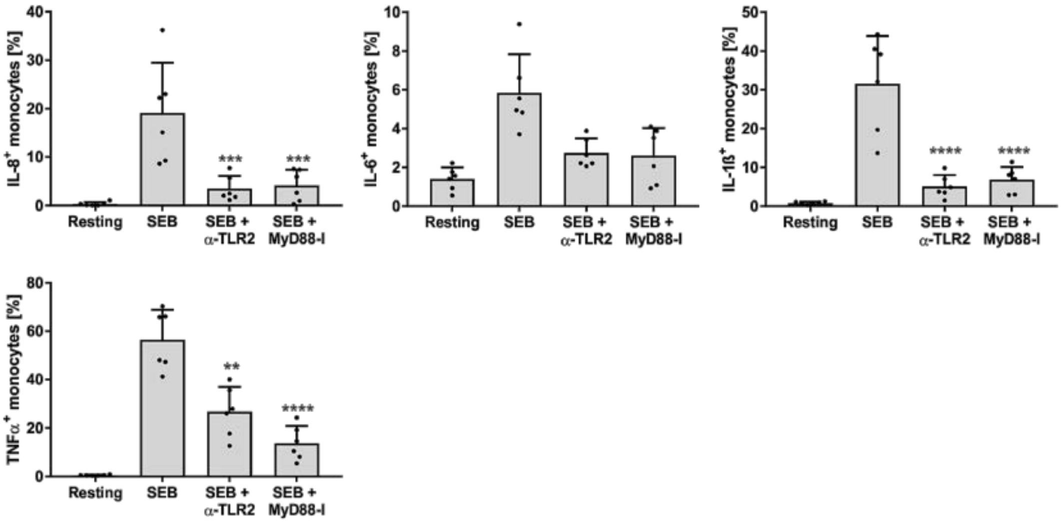

Fiure. S5. **Blockade of TLR in monocytes restricts cytokine expression**

CD14+ monocytes from adults were cultured with or without SEB in the presence or absence of the MyD88-inhibitor or anti-human TLR-2 antibody. The expression of cytokines IL-8, IL-6, IL-1ß, and TNFα was measured by flow cytometry and cumulative data from different donors was presented in bar graphs. Each dot represents a different donor. The error bars in figures denote ± SD. **p<0.01, ***p<0,001, ****p<0.0001 as determined by Kruskal Wallis test with Dunn's post hoc test.

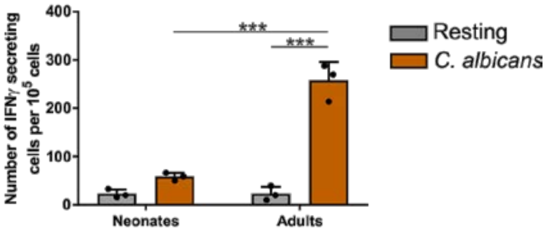

Figure S6. **ELISPOT assay for IFNγ**

Bar graph representing the ELISPOT analysis of the quantitative IFNγ produced by the $CD4^{+}CD45RA^{+}CD31^{+}$ T cells from neonates and adults which were either stimulated or not for 3 days with monocytes pulsed with *C. albicans*-lysates. Cumulative results are shown and each dot represents a different donor. The error bars in figures denote ± SD. ***$p<0.0001$ as determined by one-way Anova with Tukey post hoc test.

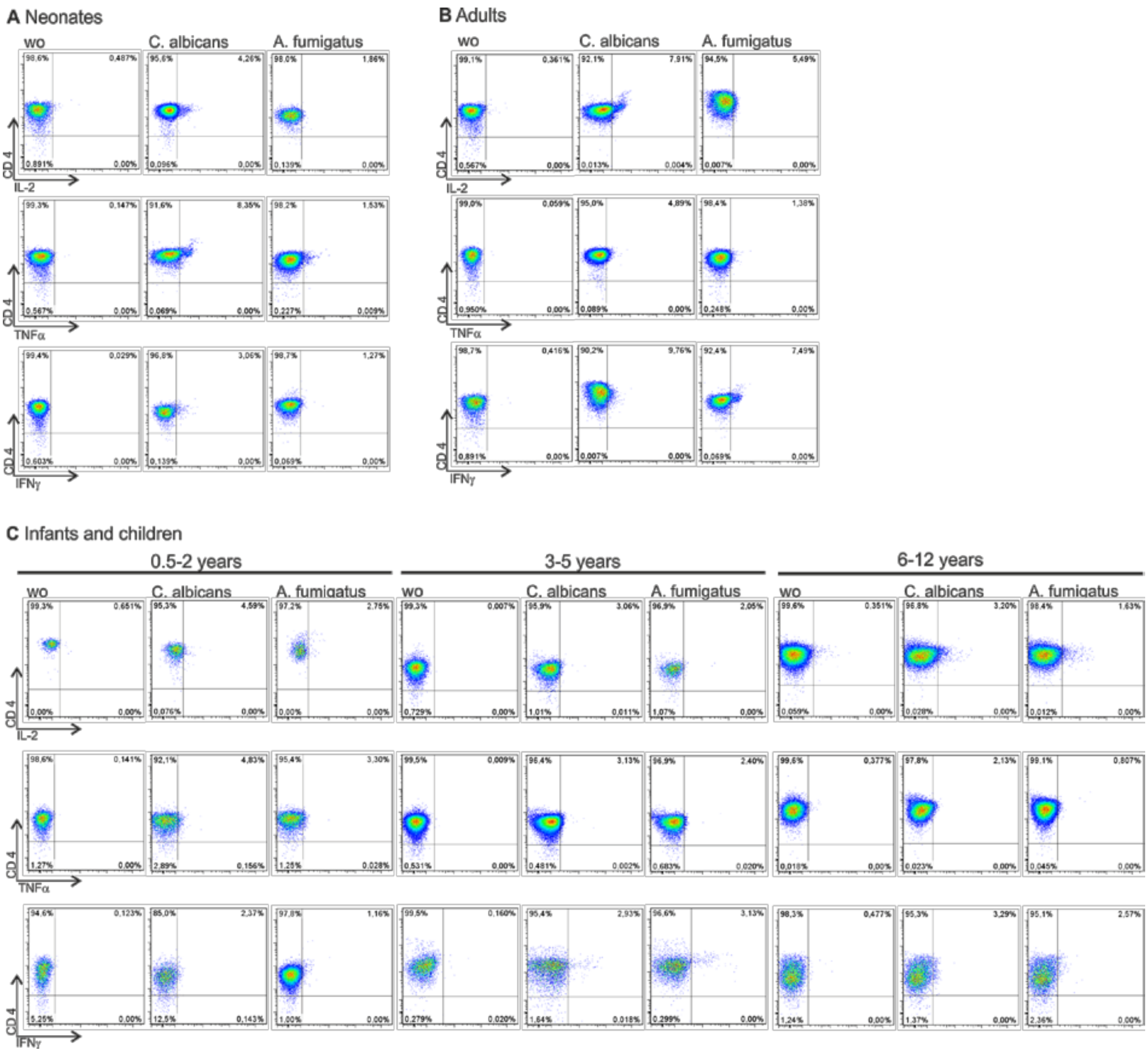

<u>Figure S7.</u> **Quantification of fungi-induced Th1 cytokine expression with age-dependent characteristics**

CD4$^+$CD45RA$^+$ T cells from neonates, infants, children, and adults were stimulated with *C. albicans* (orange) or *A. fumigatus* (blue) (as in Fig. 4) for 6 days. (**A-C**) Frequency of T cells from neonates (**A**), adults (**B**), and infants and children of different age groups (**C**), expressing intracellular IL-2, TNFα or IFNγ was determined by flow cytometry. The dot plot data is from a single donor and is a representative of the cumulative data of multiple donors shown in Fig. 4.

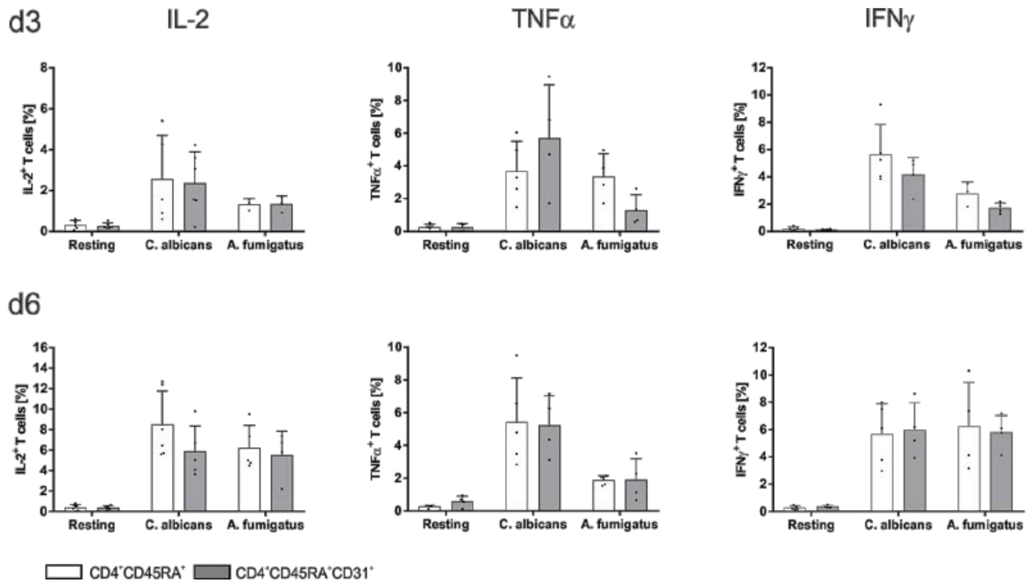

Figure S8. **Th1 cytokine expression within various T-cell subsets**

Frequency of $CD4^{+}CD45RA^{+}$ and $CD4^{+}CD45RA^{+}CD31^{+}$ T cells isolated from adult blood, expressing intracellular IL-2 (left panel), TNFα (middle panel) or IFNγ (right panel) upon stimulation for 3 and 6 days respectively, with *C. albicans or A. fumigatus* pulsed monocytes was analysed by flow cytometry. Cumulative results are shown and each dot represents a different donor. The error bars in figures denote ± SD. Data were not significantly different as determined by Kruskal Wallis test with Dunn's post hoc test.

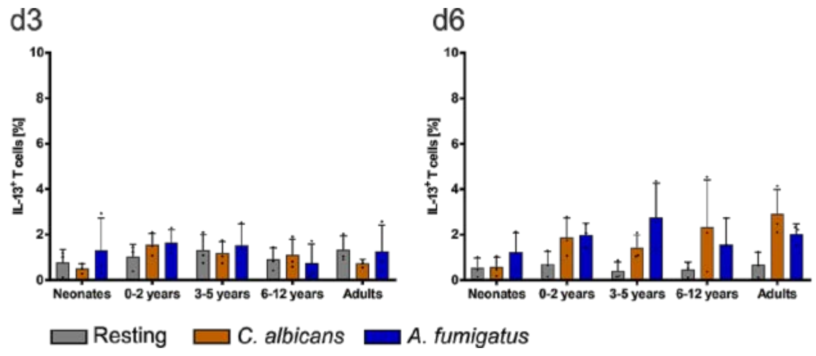

Figure S9. **Age-dependent expression of IL-13 by fungi specific T cells**

CD4+CD45RA+ T cells from neonates, infants, children, and adults were co-cultured with monocytes pulsed with *C. albicans*- or *A. fumigatus*-lysates. The frequency of T cells expressing intracellular IL-13 upon 3 days (left panel) and 6 days (right panel) after stimulation was measured by flow cytometry. Cumulative results are shown and each dot represents a different donor. The error bars in figures denote ± SD. Data were not significantly different as determined by one-way Anova with Tukey post hoc test.
